# Supplementary material for: Integrated Biobanking and Tumor Model Establishment of Human Colorectal Carcinoma Provides Excellent Tools for Preclinical Research
Source: Cancers (Basel). 2019 Oct 9;11(10):1520. doi: 10.3390/cancers11101520 (PMC6826890; doi:10.3390/cancers11101520)
Supplement: Supplementary file 1 [file cancers-11-01520-s001.zip › Legende Suppl. Data_new.docx]

| HROC | Sample ID: HRO= Hanseatic City of Rostock; C= colorectal | |
| --- | --- | --- |
| _Met | Metastasis | |
| _Rec | Local recurrence | |
| _Tu | Tumor (resection sample contained more than one tumor/patient presented with secondary, non-local recurrent tumor) | |
| UICC | Classification according to the Union Internationale Contre le Cancer | |
| n.a. | Data not available | |
| Therapy | c | Curative intent |
|  | p | Palliative intent |
|  | aRCT | Adjuvant radio chemotherapy |
|  | naRCT | Neoadjuvant radio chemotherapy |
| Recurrence | m | Metastatic recurrence |
|  | lr | Local recurrence |
| Secondary therapy | s | Surgery |
|  | ch | Chemotherapy |
|  | r | Radiotherapy |
|  | BSC | Best supportive care |
| RFS | Relapse-free survival | |
| OS | Overall survival | |
| F/U | follow-up | |
| PD | Perioperative death | |
| CIMP-H | CpG island methylator phenotype | |
| spStd | Sporadic standard type | |
| spMSI-H | Sporadic high-degree microsatellite instable type | |
| Lynch | Lynch-type | |
| PDX | Patient-derived xenograft | |
| 1 | One or more attempt to establish cell culture/PDX | |
| 2 | Successful attempt cell culture/PDX | |
| 3 | Contaminated cell culture | |
| 4 | No or not enough material available | |
| *italic* | UICC stages in italic (only for metastases) are the UICC stages of the corresponding primary CRC | |
| **bold** | Patient listed on both sheets of Supplementary Table 1 | |

The patient cohort is divided into two groups: on sheet one (= samples modelled) all patients and respective samples are listed for which tissue could successfully be collected to support at least one type of model establishment was attempted; on sheet two (= samples not modelled & NET) all patients and respective neoplasias attempted to collect are listed.
